# SK2 Channels Associate With mGlu_1α_ Receptors and Ca_V_2.1 Channels in Purkinje Cells

**DOI:** 10.3389/fncel.2018.00311

**Published:** 2018-09-19

**Authors:** Rafael Luján, Carolina Aguado, Francisco Ciruela, Xavier Morató Arus, Alejandro Martín-Belmonte, Rocío Alfaro-Ruiz, Jesús Martínez-Gómez, Luis de la Ossa, Masahiko Watanabe, John P. Adelman, Ryuichi Shigemoto, Yugo Fukazawa

**Affiliations:** ^1^Synaptic Structure Laboratory, Instituto de Investigación en Discapacidades Neurológicas (IDINE), Departamento de Ciencias Médicas, Facultad de Medicina, Campus Biosanitario, Universidad Castilla-La Mancha, Albacete, Spain; ^2^Unitat de Farmacologia, Departament de Patologia i Terapèutica Experimental, Facultat de Medicina i Ciències de la Salut, IDIBELL, Universitat de Barcelona, L’Hospitalet de Llobregat, Barcelona, Spain; ^3^Institut de Neurociències, Universitat de Barcelona, Barcelona, Spain; ^4^Department of Biochemistry and Microbiology, Faculty of Sciences, University of Ghent, Ghent, Belgium; ^5^Departamento de Sistemas Informáticos, Escuela Superior de Ingeniería Informática, Universidad de Castilla-La Mancha, Albacete, Spain; ^6^Department of Anatomy, Graduate School of Medicine, Hokkaido University, Sapporo, Japan; ^7^Vollum Institute, Oregon Health and Science University, Portland, OR, United States; ^8^Institute of Science and Technology (IST Austria), Klosterneuburg, Austria; ^9^Division of Brain Structure and Function, Research Center for Child Mental Development, Life Science Advancement Program, Faculty of Medical Science, University of Fukui, Fukui, Japan

**Keywords:** cerebellum, potassium channel, mGlu receptor, electron microscopy, immunohistochemistry, calcium channel, synapse

## Abstract

The small-conductance, Ca^2+^-activated K^+^ (SK) channel subtype SK2 regulates the spike rate and firing frequency, as well as Ca^2+^ transients in Purkinje cells (PCs). To understand the molecular basis by which SK2 channels mediate these functions, we analyzed the exact location and densities of SK2 channels along the neuronal surface of the mouse cerebellar PCs using SDS-digested freeze-fracture replica labeling (SDS-FRL) of high sensitivity combined with quantitative analyses. Immunogold particles for SK2 were observed on post- and pre-synaptic compartments showing both scattered and clustered distribution patterns. We found an axo-somato-dendritic gradient of the SK2 particle density increasing 12-fold from soma to dendritic spines. Using two different immunogold approaches, we also found that SK2 immunoparticles were frequently adjacent to, but never overlap with, the postsynaptic density of excitatory synapses in PC spines. Co-immunoprecipitation analysis demonstrated that SK2 channels form macromolecular complexes with two types of proteins that mobilize Ca^2+^: Ca_V_2.1 channels and mGlu_1α_ receptors in the cerebellum. Freeze-fracture replica double-labeling showed significant co-clustering of particles for SK2 with those for Ca_V_2.1 channels and mGlu_1α_ receptors. SK2 channels were also detected at presynaptic sites, mostly at the presynaptic active zone (AZ), where they are close to Ca_V_2.1 channels, though they are not significantly co-clustered. These data demonstrate that SK2 channels located in different neuronal compartments can associate with distinct proteins mobilizing Ca^2+^, and suggest that the ultrastructural association of SK2 with Ca_V_2.1 and mGlu_1α_ provides the mechanism that ensures voltage (excitability) regulation by distinct intracellular Ca^2+^ transients in PCs.

## Introduction

Purkinje cells (PCs) are fundamental to the function in the cerebellum, as out of the different neuron populations they are the only ones projecting out of the cerebellar cortex (Altman and Bayer, 1997). The activity of PCs is controlled both by topographically organized excitatory and inhibitory inputs to somato-dendritic domains and by intrinsic conductances that cause their spontaneous firing (Häusser and Clark, 1997; Jaeger and Bower, 1999; Swensen and Bean, 2003). Ion channels operating along the plasma membrane of PCs largely determine the generation, patterns and propagation of electrical signals, which are required for the neuronal intrinsic activity. One type of ion channel controlling spontaneous activity in PCs is the small conductance Ca^2+^-activated K^+^ (SK) channels (Womack and Khodakhah, 2003).

SK channels are atypical in the sense that they are voltage-independent and activated only by cytosolic Ca^2+^ (Kohler et al., 1996; Ngo-Anh et al., 2005; Luján et al., 2009). There are three mammalian SK channel genes (SK1, SK2 and SK3) and the three are expressed in the cerebellum, where they show different cellular profiles. At the cellular level, all three subtypes are expressed in granule cells, but only SK2 is expressed in PCs (Cingolani et al., 2002; Sailer et al., 2002; Gymnopoulos et al., 2014). At the functional level, SK2 channels are known to modulate Ca^2+^ transients and to influence spike-firing frequency in dendritic spines, as well as contributing to activity-dependent and compartment-specific plasticity in dendrites of PCs (Hosy et al., 2011; Ohtsuki et al., 2012; Grasselli et al., 2016). When SK channels are down-regulated, the enhanced Ca^2+^ transients taking place in spines result in a lower probability in LTP induction (Belmeguenai et al., 2010; Hosy et al., 2011). The way SK channels regulate these forms of plasticity most likely arises from their specific location along the neuronal surface, as well as from their interaction with different proteins mobilizing Ca^2+^. SK2 channels are located at synaptic and extrasynaptic sites in the hippocampus, where they interact with NMDA and mGlu_5_ receptors, respectively (Lin et al., 2008; Ballesteros-Merino et al., 2012; García-Negredo et al., 2014). However, PCs do not express these receptors, suggesting that SK2 channels are associated with other group of molecules controlling cytosolic Ca^2+^ transients, including Ca_V_2.1 channels (Womack and Khodakhah, 2003) and mGlu_1α_ receptors (ref.) that show high levels of expression in PCs (Starr et al., 1991). However, the association between SK channels and the sources of Ca^2+^ for their activation in the cerebellar cortex remains unexplored.

Recent immunoelectron microscopic analysis in the developing cerebellum showed that SK2 channels progressively accumulate with age along the extrasynaptic plasma membrane of PC spines. However, due to the technical limitations associated with the pre-embedding immunogold method in the detection of intrasynaptic proteins (Luján, 2010), convincing evidence for the location of SK2 channels along the surface of PCs awaits the use of more sensitive approaches.

To address these questions, we have exploited the freeze-fracture replica immunogold labeling (SDS-FRL) technique, a new quantitative, immunoelectron microscopic method of high sensitivity (Masugi-Tokita and Shigemoto, 2007; Luján et al., 2018), to determine the precise subcellular distribution and densities of SK2, and the spatial relationship between SK2 with Ca_V_2.1 channels and mGlu_1α_ receptors, along the neuronal surface of PCs. In addition, we probed for the possible formation of macromolecular complexes between SK2 channels, Ca_V_2.1 channels and mGlu1α receptors using co-immunoprecipitation.

## Materials and Methods

### Animals

Adult C57BL/6J mice (*n* = 3) obtained from the Animal House Facility of the National Institute for Physiological Sciences (NIPS, Okazaki, Japan) were used in this study for SDS-FRL techniques, and adult C57BL/6J mice (*n* = 3) obtained from the Animal House Facility of the University of Castilla-La Mancha (Albacete, Spain) were used for post-embedding immunoelectron microscopic approaches. For Co-IP, adult C57BL/6J mice (*n* = 4) obtained from the Animal House Facility of the Universitat de Barcelona. In addition, we used wild type (*n* = 3) and SK2 knockout mice (*n* = 3) from the Vollum Institute (Cueni et al., 2008; Lin et al., 2008). Care and handling of animals prior to and during experimental procedures was in accordance with Japanese, USA and European Union regulations (86/609/EC), and the protocols were approved by the local Animal Care and Use Committee (CEEA) of the University of Castilla-La Mancha (Albacete, Spain).

### Antibodies and Chemicals

**Figure 1:**
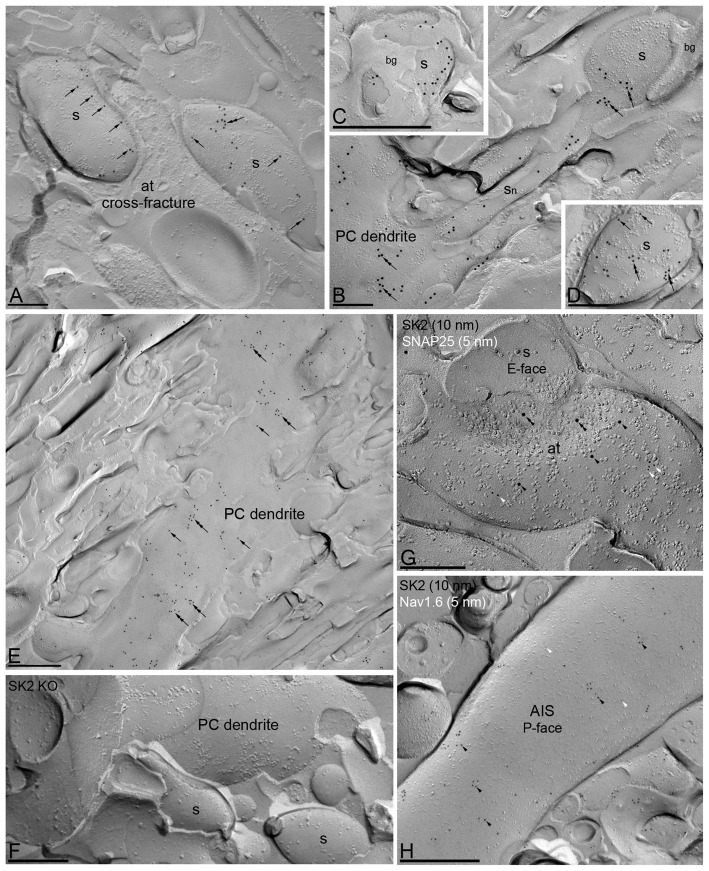
Subcellular localization of SK2 channels along the surface of Purkinje cells (PCs) using the SDS-digested freeze-fracturereplica labeling (SDS-FRL) technique. Immunolabeling is restricted to the plasma membrane P-face of PCs. The E-face of Bergmann glia (bg), cross-fractures (panel **A**) or E-faces of neurons (panel **G**) are free of any immunolabeling. **(A–E)** Immunoparticles for SK2 channels (10 nm gold) are scattered (arrows) and clustered (double arrows) in dendritic spines (S) and dendritic shafts (PC dendrite, DEN) of PCs. **(G)** The image shows a high-magnification of the replicated plasma membrane of an axon terminal (at), presumably from granule cells, and spines (s) in a E-face view, showing the co-localization of SK2 (10 nm immunogold) and SNAP25 (5 nm immunogold, white arrowheads). Within this presynaptic localization, SK2 immunoparticles were observed both along the active zone (AZ; double black arrowheads) and at extrasynaptic membranes (black arrowheads). **(H)** The image shows the P-face of an axon initial segment (AIS) co-labeled for the SK2 (10 nm, black arrowheads) and Nav1.6 (5 nm, white arrowheads) channels. Very low frequency of immunogold labeling for SK2 was detected along AIS. **(F)** The specificity of the antibody was confirmed in replicas of SK2 channel-knockout mice (SK2 KO) that were free of any immunolabeling. Scale bar: **(A,D,F,G)** 0.2 μm; **(E,H)** 0.5 μm.

Table 1 shows a complete list of the primary antibodies, together with their source, dilution, characteristics and specificity that were used in this study. This work also provided additional information about the specificity of the anti-SK2 antibodies using immunohistochemical techniques in the cerebellum (Figure 1F). Secondary antibodies conjugated to 5 nm or 10 nm gold particles were purchased from British Biocell International (BBI, Cardiff, UK).

**Table 1:** Identity, source and characterization of antibodies.

Molecule	Code, Reference	Host Animal	Developer, Supplier	Epitope aa sequence	Protein concentration	Optimal Dilution FRIL	Reference, production, characterization
SK2	GP-Af540	Guinea pig	M. Watanabe	Mouse aa 536–574	0.2 mg/mL	1:60	Ballesteros-Merino et al. (2012)
SK2	Rb-Af500	Rabbit	M. Watanabe	Mouse aa 536–574	0.2 mg/mL	1:100	Lin et al. (2008); This article
Cav2.1	GP-Af810	Guinea pig	M. Watanabe	Mouse aa 361–400	0.2 mg/mL	1:90	Indriati et al. (2013)
mGluR1α	A53	Rabbit	R. Shigemoto	Rat aa 859–1199	0.54 mg/mL	1:1,000	Shigemoto et al. (1997)
mGluR1α	GP-Af660	Guinea pig	M. Watanabe	Mouse aa 945–1127	0.2 mg/mL	1:100	Nakamura et al. (2004)
Gluδ2	Rb-Af500–1	Rabbit	M. Watanabe	Mouse aa 852–931	0.2 mg/mL	1:250	Konno et al. (2014)
SNAP-25	Ref 111 002	Rabbit	Synaptic Systems	Human aa 192–206	0.2 mg/mL	1:1,500	https://www.sysy.com
Nav1.6	K87A/10	Mouse	NeuroMab	Rat aa 459–476	—	1:100	http://neuromab.ucdavis.edu
Calbindin	300	Mouse	Swant	—	supernatant	—	https://www.swant.com/

*aa: amino acid*.

### Co-immunoprecipitation

Mice were sacrificed by decapitation and then the cerebellum was carefully dissected. Once the tissue samples were kept in a chilled eppendorf, ice-cold Tris 50 mM buffer containing the Protease Inhibitor Cocktail was added. Then, the tissue was homogenized with the polytron for three periods of 10 s each. Next, in order to remove the nucleus and tissue debris, we spin the homogenate for 10 min at 1,000× *g* at 4°C. The supernatant containing the membrane extracts was recovered in a new eppendorf and centrifuge for 30 min at 12,000× *g* at 4°C. The supernatant containing the cytosolic proteins was discarded and the pellet containing the membrane extracts was resuspended in 1 mL of Tris 50 mM with Protease Inhibitor Cocktail. Membrane extracts were solubilized with radio immunoprecipitation assay (RIPA) buffer (50 mM Tris-HCl (pH 7.4), 100 mM NaCl, 1% Triton-X100, 0.5% sodium deoxycholate, 0.2% SDS and 1 mM EDTA) during 30 min on ice. The solubilized extract was then centrifuged at 13,000 × *g* for 30 min. The supernatant (1 mg/ml) was processed for immunoprecipitation. These steps were conducted with constant rotation at 0–4°C. Next, we incubated overnight the supernatant with anti-Cav2.1, anti-SK2 or anti-mGluR1α polyclonal antibodies. All further steps were performed according to the procedure described recently (Luján et al., 2018).

### Immunohistochemistry for Electron Microscopy

Immunohistochemical reactions at the electron microscopic level were carried out using the post-embedding immunogold and SDS-FRL techniques as described previously (Luján et al., 1996, 2018; Tanaka et al., 2005). All ultrastructural analyses were carried out in a JEOL-1010 transmission electron microscope.

#### Post-embedding Immunogold Method

Animals (*n* = 3 mice) were anesthetized by intraperitoneal injection of ketamine-xylazine 1:1 (0.1 mL/kg b.w.) and transcardially perfused with ice-cold fixative containing 4% paraformaldehyde, 0.1% glutaraldehyde and 15% saturated picric acid solution in 0.1 M phosphate buffer (PB) for 15 min. Coronal sections of 500 μm thickness were obtained using a vibratome. Sections were placed into 1 M sucrose solution in 0.1 M PB for 2 h and then they were plunge frozen by a Leica EM CPC apparatus. Samples were dehydrated in methanol at −80°C and embedded by freeze-substitution (Leica EM AFS2) in Lowicryl HM 20 (Electron Microscopy Science, Hatfield, USA), followed by polymerization with UV light. Then, ultrathin 80-nm-thick sections from Lowicryl-embedded blocks of the hippocampus were picked up on coated nickel grids and incubated on drops of a blocking solution consisting of 2% human serum albumin in 0.05 M TBS and 0.03% Triton X-100. The grids were incubated with SK2 antibodies (10 μg/mL in 0.05 M TBS and 0.03% Triton X-100 with 2% human serum albumin) at 28°C overnight. The grids were incubated on drops of goat anti-rabbit IgG conjugated to 10 nm colloidal gold particles (Nanoprobes Inc.) in 2% human serum albumin and 0.5% polyethylene glycol in 0.05 M TBS and 0.03% Triton X-100. The grids were then washed in TBS and counterstained for electron microscopy with 1% aqueous uranyl acetate followed by Reynolds’s lead citrate.

#### SDS-Digested Freeze-Fracture Replica Labeling (SDS-FRL) Technique

SDS-FRL was performed with some modifications (Tanaka et al., 2005; Luján et al., 2018) to the original method described by Fujimoto (1995). Animals (*n* = 3 mice) were anesthetized by intraperitoneal injection of ketamine-xylazine 1:1 (0.1 mL/kg b.w.) and transcardially perfused with ice-cold fixative containing 2% paraformaldehyde and 15% saturated picric acid solution in 0.1 M PB for 12 min. The cerebella were dissected and the vermis was cut into sagittal slices (130 μm) using a Microslicer (Dosaka, Kyoto, Japan) in 0.1 M PB. All further steps employed to obtain replicas were performed according to the protocol described recently (Luján et al., 2018). The replicas were washed and reacted with a polyclonal rabbit antibody for SK2 (8 μg/mL) overnight at 15°C overnight. Following washes and blocking in 5% BSA/TBS, replicas were incubated in secondary antibodies conjugated with 10-nm gold particles overnight at room temperature. When the primary antibody was omitted, no immunoreactivity was observed. For double labeling of SK2 with Ca_V_2.1, replicas were first reacted with the SK2 antibody (8 μg/mL) and then anti-rabbit secondary antibody, followed by incubation with the Ca_V_2.1 (8 μg/mL) antibody and appropriate anti-guinea pig secondary antibody. For double labeling of SK2 with mGlu_1α_, replicas were first reacted with the SK2 antibody (8 μg/mL) and then anti-guinea pig secondary antibody, followed by incubation with the mGlu_1α_ (8 μg/mL) antibody and appropriate anti-rabbit secondary antibody. For double labeling of mGlu_1α_ with Ca_V_2.1 replicas were first reacted with the mGlu_1α_ antibody (8 μg/mL) and then anti-rabbit secondary antibody, followed by incubation with the Ca_V_2.1 (8 μg/mL) antibody and appropriate anti-guinea pig secondary antibody. After immunogold labeling, replicas were rinsed and picked up onto 100-mesh copper coated with pioloform (Agar Scientific, Stansted, Essex, UK).

### Quantification and Analysis of SDS-FRL Data

The labeled replicas were photographed at magnifications of 60,000, 80,000 and 100,000. All antibodies that were employed in the present study were visualized by immunoparticles on the protoplasmic face (P-face), consistent with the intracellular location of their epitopes. Non-specific background labeling was measured on the exoplasmic face (E-face) in WT mice and both on E-face and on P-face of the SK2 KO mice.

#### Density Gradient of SK2 Channels Along the Neuronal Surface

Quantitative analysis of immunogold labeling for SK2 was performed on six different dendritic compartments of PCs in the outer 2/3 and the inner 1/3 of the ML (the innervation territories of stellate and basket cell, respectively), in PC somata in the PC layer and the axon initial segments (AISs) in the upper part of the granule cell layer. The dendritic compartments analyzed were the main dendritic shaft, spiny branchlets (oblique dendrites) and dendritic spines. The ultrastructural criteria used to identify these neuronal compartments were described elsewhere (Luján et al., 2018). Non-specific labeling was measured on E-face structures close to the analyzed P-faces. To quantify the density of SK2 channels in the AISs, Na_V_1.6 was used as a molecular marker. Images of the identified PC compartments were selected randomly over the entire dendritic tree of PCs and then captured with an ORIUS SC1000 CCD camera (Gatan, Munich, Germany). Digitized images were modified for brightness and contrast using Adobe PhotoShop CS5 (Mountain View, CA, USA). The area of the selected profiles and the number of immunoparticles were calculated using the GPDQ software, which was described in detail recently (Luján et al., 2018).

#### Analysis of the Spatial Associations of SK2 Channels, Ca_V_2.1 Channels and mGlu_1α_ Receptors

At postsynaptic sites, nearest neighbor distances (NNDs) from SK2 to Ca_V_2.1 particles, from SK2 to mGlu_1α_ particles, and from mGlu_1α_ to Ca_V_2.1 particles were measured. At presynaptic sites, NNDs between SK2 and Ca_V_2.1 were measured using the GPDQ software. All further steps used to measure the distances between two particles, comparisons with intra-type NNDs and generation of simulations were performed similarly to the procedure described recently (Luján et al., 2018).

### Controls

Method specificity in the procedures for electron microscopy were tested. In such case, the primary antibody was either omitted or replaced with 5% (v/v) normal serum of the species of the primary antibody, resulting in total loss of the signal. For the post-embedding technique, labeling patterns obtained by the SK2 antibody were also compared with those obtained by the Calbindin antibody (polyclonal rabbit anti-Calbindin D-9k CB9; Swant, Marly, Switzerland); only the antibodies against SK2 consistently labeled the plasma membrane. For double-labeling in SDS-FRL approaches, any possible cross-reactivity of secondary antibodies was tested by incubating some replicas with only one primary antibody and the full complement of the secondary antibodies. No cross-labeling that would influence the results was detected. In addition, some replicas were incubated with the two primary antibodies, but we swapped the size of immunogold in the secondary antibodies for the two targets proteins. No differences were detected in measured distances that would influence our results. Furthermore, antiserum against SK2 was tested on replicas prepared from SK2 KO mice. The immunosignal was totally missing in those cerebellar areas where a strong signal was observed in control sections (Figure 1F).

### Data Analysis

Statistical analyses for morphological data were performed using appropriate software (GraphPad Prism 5, La Jolla, CA, USA) and data were presented as mean ± SEM unless indicated otherwise. Statistical significance was defined as *p* < 0.05. The statistical evaluation of the immunogold densities was performed using the Kruskal–Wallis test, pairwise Mann–Whitney *U* test and Dunns method. Correlations were assessed using Pearson’s correlation test. To assess colocalization between receptor and ion channels for each compartment, two-sided paired *t*-test followed by Holm-Bonferroni correction for multiple testing was used.

## Results

### Immunoreactivity for SK2 Channels Is Non-uniform in Purkinje Cells

The pre-embedding immunogold method allowed us to report that SK2 channels were widely distributed in developing and adult PCs (Ballesteros-Merino et al., 2014a). To visualize more accurately the 2D distribution of SK2 along distinct axo-somato-dendritic compartments of mature PCs and to analyze channel densities quantitatively, the SDS-FRL method (Fujimoto, 1995) was used in this study. We systematically investigated immunogold particle densities in main dendritic shafts, spiny branchlets and dendritic spines in both the inner 1/3 and outer 2/3 part of the molecular layer, in addition to the somata of PCs in the PC layer and the AIS of PCs in the granule cell layer. Electron microscopic analysis of the replicas revealed immunogold labeling for SK2 channels on P-faces of plasma membranes, in agreement with the intracellular location of the epitope (Table 1) recognized by the rabbit anti-SK2 antibody. In PCs, immunoparticles for SK2 channels were detected along the soma, dendritic shafts and dendritic spines including the spine neck, either clustered or scattered (Figure 1). The clustered pattern consists of aggregation of immunoparticles (>3 gold particles) with <50 nm distance from the closest immunoparticle and the scattered pattern consists of isolated single immunoparticle detected along the neuronal surface (Figures 1A–E). Virtually no labeling was observed on the E-face or on the cross-fractures (Figures 1A–E). Specificity of SK2 immunolabeling using the SDS-FRL technique was confirmed in cerebellar tissue of SK2 KO mice (Figure 1F), where immunogold particles were mostly abolished (SK2 KO, 0.63 ± 0.03 particles/μm^2^, WT mice, 51.23 ± 2.78 particles/μm^2^).

Immunoparticles for SK2 were not only detected in somato-dendritic domains of PCs, but were also confined to putative presynaptic axon terminals (ATs). To unmistakably identify the immunolabeled compartments, double labeling for SK2 and SNAP-25, a member of the SNARE complex present exclusively in axons, were performed (Hagiwara et al., 2005). In SNAP-25-immunopositive profiles, immunoparticles for SK2 were detected at the presynaptic active zone (AZ) and at extrasynaptic sites of ATs, likely parallel fiber (PF) terminals (Figure 1G). Finally, double-labeling experiments for SK2 and Nav1.6 revealed very low expression of SK2 channels along AISs of PCs (Figure 1H).

**Figure 2:**
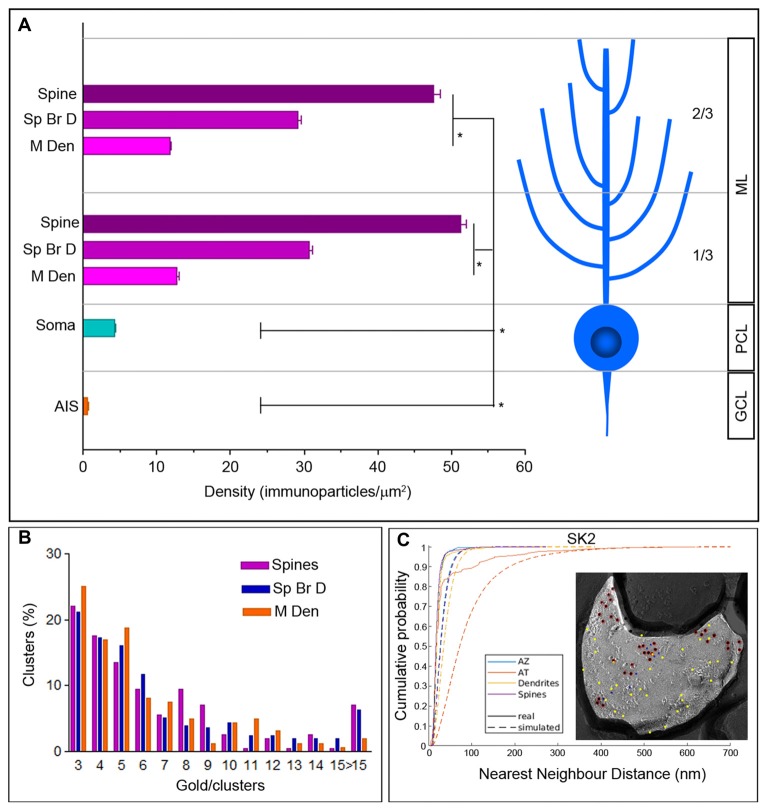
Density gradient of SK2 immunoparticles in the surface of PCs. **(A)** Bar graph shows the density (mean ± SEM) of the SK2 channels in eight compartments of PCs. Density of SK2 immunoparticles increased from soma to dendritic spines (soma = 4.31 ± 0.13/μm^2^; 1/3 ML main dendrite = 12.69 ± 1.96/μm^2^; 2/3 ML main dendrite = 11.77 ± 0.81/μm^2^; 1/3 ML spiny branchlet dendrite = 30.67 ± 3.11/μm^2^; 2/3 ML spiny branchlet dendrite = 29.14 ± 2.69/μm^2^; 1/3 ML spines = 51.23 ± 2.78/μm^2^; 2/3 ML spines = 47.60 ± 2.69/μm^2^; Kruskal–Wallis test, pairwise Mann–Whitney *U* test and Dunns method, *p < 0.001). Immunoparticle density for SK2 channels on the AISs was not significantly different from the background (AIS = 0.64 ± 0.02/μm^2^; background = 0.62 ± 0.03/ μm^2^). ML, molecular layer; PCL, Purkinje cell layer; GCL, granule cell layer. **(B)** The graph shows the quantification for the number of SK2 particles per cluster in the spines, spiny branchlets (Sp Br D) and main dendrites (M Den). Approximately 76% of clusters in dendritic spines and spiny branchlets and 81% in main dendrites were in the range of 3–8 immunoparticles. **(C)** Cumulative probability plots of SK2 to SK2 NND. Real and simulated SK2 are shown by solid and dotted lines, respectively. AZ, active zone; AT, axon terminal. The EM image is an example that show random simulation of SK2 immunoparticles in a dendritic spine. Red, real SK2; Yellow, simulated SK2; blue, real Cav2.1.

Next, to investigate whether SK2 shows a compartmentalized distribution, seven somato-dendritic compartments of PCs obtained from single labeling experiments and one axonal compartment obtained from double labeling experiments were investigated. Then, the average immunoparticle densities in each neuronal domain were calculated. We found an increase in the density of immunoparticles for SK2 going from the soma to dendritic spines (Figure 2A; Table 2). Thus, dendritic spines of PCs had 12 times higher density of immunoparticles for SK2 than soma, four times higher than apical dendrites and 1.7 times higher than spiny branchlets (Figure 2A; *p* < 0.001 for soma vs. dendritic spines; *p* = 0.003 for dendritic spines vs. spiny branchlets; *p* = 0.003 for spiny branchlets vs. main dendrites, Kruskal–Wallis test, pairwise Mann–Whitney *U* test and Bonferroni correction). No differences were detected in the density of SK2 in the dendritic compartments between the 1/3 and 2/3 of the molecular layer (Figure 2A; Table 2). Finally, we also evaluated the density of SK2 channels in AISs of PCs, calculated from double labeling experiments with the Nav1.6 subunit. AISs contained 0.64 ± 0.02 immunoparticles/μm^2^, which did not differ significantly from the background (0.63 ± 0.03 immunoparticles/μm^2^; *P* = 0.91, Mann–Whitney *U* test; Figure 2A; Table 2).

**Table 2:** Densities of immunoparticles labeling the SK2 subunit in different axo-somato-dendritic compartments of Purkinje cells (PCs).

Mouse	BG	AIS	Soma	Prox M Den	Dist M Den	Prox Sp Br Den	Dist Sp Br Den	Prox Sp	Dist Sp
#1	0.71	0.63 (10, 11)	3.82 (10, 102)	13.25 (10, 629)	10.06 (10, 252)	37.24 (14, 720)	13.01 (14, 378)	46.83 (20, 210)	47.68 (20, 250)
#2	0.63	0.67 (10, 11)	4.71 (10, 113)	11.44 (10, 302)	11.66 (10, 287)	19.08 (14, 373)	37.16 (14, 491)	53.18 (20, 199)	40.80 (20, 168)
#3	0.54	0.62 (10, 10)	4.39 (10, 116)	13.41 (10, 286)	13.59 (10, 380)	35.69 (14, 534)	26.60 (14, 509)	53.69 (20, 208)	54.33 (20, 206)
Mean	**0.63**	**0.64**	**4.31**	**12.70**	**11.77**	**30.67**	**29.14**	**51.23**	**47.60**
SEM	0.03	0.02	0.13	1.96	0.81	3.11	2.69	2.78	2.69

*Mean density values are provided in immunogold/μm^2^. In parenthesis, the first number indicates the number of analyzed profiles and the second number denotes the number of counted gold particles*.

We next compared the NNDs between real and simulated SK2 immunoparticles that allowed us to analyze if SK2 channels are significantly clustered or not. First, we compared the NNDs between real and simulated SK2 immunoparticles from all images and found a significant clustering in dendritic spines, dendritic shafts and AZ (Figure 2C, *P* < 0.001 for all compartments). Next, we analyzed the existence of significant clustering on individual images. For that purpose, we compared the mean NND of real SK2 immunoparticles in each image with those of randomly distributed particles with *uniform probability* and judged the image to show a significant association if the real mean NND was within the smallest 2.5% of simulated mean NNDs or a significant dissociation if the real mean NND was within the largest 2.5% of simulated mean NNDs. We observed in dendritic spines that 96.5% of profiles showed a significant association of SK2 immunoparticles with each other (*n* = 57), indicating significantly clustered distribution of SK2. In dendritic shafts (*n* = 25) and AZs (*n* = 29), 100% of profiles showed a significant association of SK2 immunoparticles with each other.

We further analyzed the number of immunogold particles per cluster at different dendritic compartments (Figure 2B). First, we fixed the minimum number of immunoparticles in a cluster to three, which means that single or pairs of particles were discarded for clustering classification. Thus, a given pair of immunoparticles at distance smaller than or equal to the minimum inter-cluster distance threshold belongs to the same cluster. We obtained the value of the threshold parameter from the distribution of the distances between each immunoparticle and its nearest neighbor immunoparticle, using mean + two times the standard deviation of such distances. Following application of these parameters to our data, we found that the number of immunoparticles per cluster was similar between spines, spiny branchlets and main dendrites and around 76% of clusters in dendritic spines and spiny branchlets, and 81% in main dendrites, contained three to eight immunoparticles (Figure 2B).

### SK2 Channels Are Excluded From PSDs in Purkinje Cell Spines

**Figure 3:**
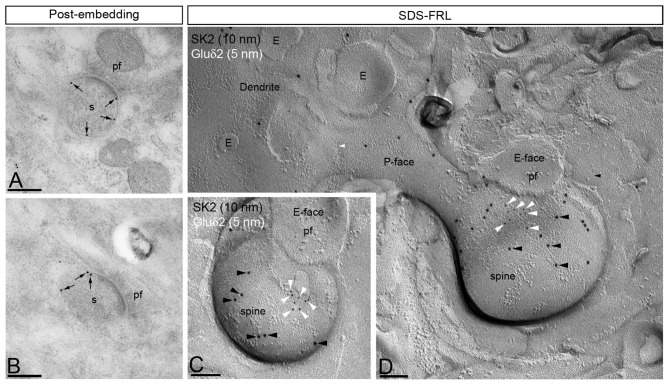
SK2 channels are excluded from the postsynaptic membrane specializations in PC spines. Electron micrographs showing immunoparticles for SK2 in the molecular layer of the cerebellar cortex, as detected using the post-embedding immunogold (panels **A,B**) and the SDS-FRL (panels **C,D**) techniques in the adult mice. **(A,B)** Using the post-embedding immunogold method, immunoparticles for SK2 were always detected outside the synaptic specialization and only detected both at perisynaptic and extrasynaptic sites (arrows) of PC spines (s) establishing excitatory synapses with parallel fiber terminals (pf). **(C,D)** Using the highly sensitive SDS-FRL technique, immunoparticles for SK2 (10 nm, black arrowheads) did not co-cluster with particles for Gluδ2 that is expressed selectively at PF-PC synapses (5 nm, white arrowheads), demonstrating the absence of SK2 from the PSD of excitatory synapses between PC spines and PFs. Scale bars: **(A,B)** 0.2 μm; **(C,D)** 0.1 μm.

The presence of SK2 has been reported along the PSD of excitatory synapses in the hippocampus (Lin et al., 2008; Ballesteros-Merino et al., 2012) and ventral tegmental area (VTA; Soden et al., 2013). Here, we investigated if SK2 was also located at PSD in PF-PC synapses using the post-embedding immunogold technique (Figures 3A,B). Immunoparticles for SK2 were always observed at perisynaptic or extrasynaptic sites in PC spines establishing synapses with PFs, but never in the main body of the postsynaptic membrane specialization (Figures 3A,B). To further corroborate our post-embedding immunoelectron microscopic data we carried out SDS-FRL experiments. However, cluster of IMPs identifying glutamatergic synapses on the replica E-face cannot be seen on the P-face. For this reason, we performed double-labeling for SK2 and the delta 2 glutamate receptor (Gluδ2), which represents a P-face marker for PF-PC synapses (Figures 3C,D). The Gluδ2 is selectively expressed within the main body of PF–PC synapses (Araki et al., 1993). This experiment confirmed that immunoparticles for SK2 were always segregated from clusters of Gluδ2 immunoparticles (Figures 3C,D), indicating a lack of association between SK2 channels and synaptic specializations.

### SK2 Forms Macromolecular Complexes With Ca_V_2.1 and mGlu_1α_ in Cerebellar Membranes

**Figure 4:**
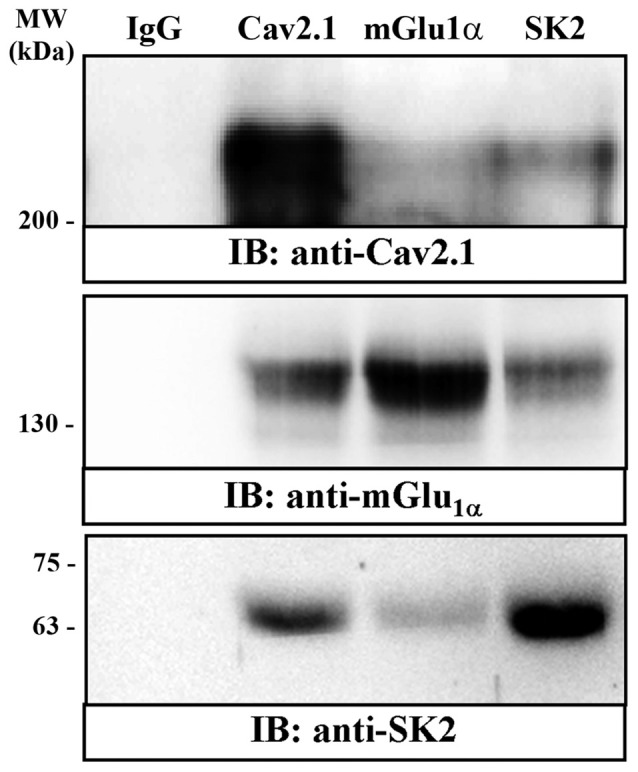
Co-immunoprecipitation of mGlu_1α_ receptor and SK2 and Cav2.1 channels from mouse cerebellum. Solubilized cerebellar membrane extracts underwent immunoprecipitation analysis using rabbit control IgG (2 μg, lane 1); rabbit anti-Cav2.1 (2 μg, lane 2); rabbit anti-mGlu_1α_ (2 μg, lane 3) and rabbit anti-SK2 (2 μg, lane 4). Immunoprecipitates (IP) were investigated by SDS-PAGE and immunoblotted using a rabbit anti-Ca_V_2.1 (1 μg/ml), rabbit anti-mGlu_1α_ (1 μg/ml) and guinea-pig anti-SK2 (1 μg/ml). Immunoreactive bands were detected as we have described in detail in the experimental procedures.

To evaluate the physiological relevance of the SK2 channel, Ca_V_2.1 channel and mGlu_1α_ receptor interaction, co-immunoprecipitation was performed in mouse cerebellum. Therefore, using soluble extracts from mouse cerebellum, the anti-Cav2.1, the anti-mGlu_1α_ and the anti-SK2 antibodies were able to immunoprecipitate a band of ~250 kDa (Figure 4, lane 2, IP: anti-Cav2.1), ~145 kDa (Figure 4, lane 3, IP: anti-mGlu_1α_) and ~65 kDa (Figure 4, lane 4, IP: anti-SK2) which correspond to the Cav2.1 channel, the mGlu_1α_ receptor and the SK2 channel, respectively. Interestingly, the anti-Cav2.1 antibody was able to co-immunoprecipitate the mGlu_1α_ receptor (Figure 4: lane 2, IB: anti-mGlu_1α_), as previously reported (Kitano et al., 2003) and the SK2 channel (Figure 4: lane 2, IB: anti-SK2). In addition, the anti-mGlu_1α_ antibody was also able to faintly co-immunoprecipitate the Cav2.1 channel (Figure 4: lane 3, IB: anti-Cav2.1) and the SK2 channel (Figure 4: lane 3, IB: anti-SK2). Conversely, anti-SK2 antibody co-immunoprecipitated the Cav2.1 channel and the mGlu_1α_receptor (Figure 4, lane 4, IB: anti-Cav2.1and IB: anti-mGlu_1α_, respectively). Importantly, the bands were not present when we used an irrelevant guinea pig or rabbit IgG for immunoprecipitation (Figure 4, lane 1), showing that the reaction was specific. Overall, the present data suggest that in mouse cerebellum SK2 channel, Ca_V_2.1 channel and mGlu_1α_ receptor might assemble into stable protein-protein complexes. This makes reasonable the idea that these oligomeric complexes might be of physiological importance *in vivo*.

### Co-localization of SK2 With Ca_V_2.1 and mGlu_1α_ in PC Spines and Dendrites

**Figure 5:**
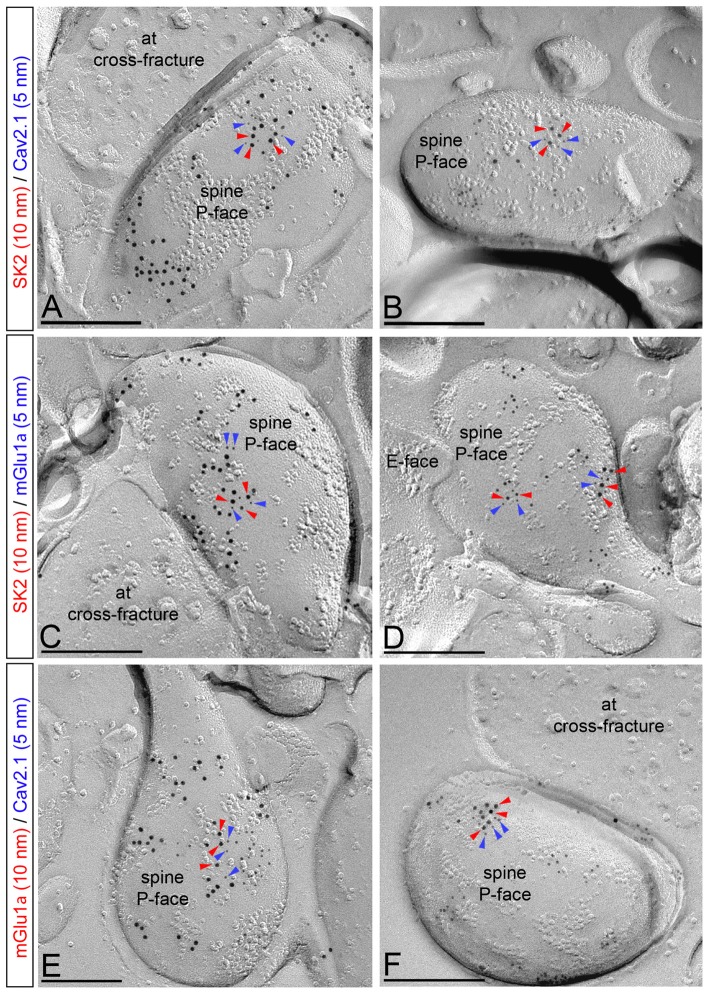
Co-localization of SK2 channels with Ca_V_2.1 channels and mGlu_1α_ receptor in PC spines. Electron micrographs obtained in the molecular layer of the cerebellar cortex showing double labeling for SK2 (10 nm, red arrowheads) and Ca_V_2.1 (5 nm, blue arrowheads; panels **A,B**), double labeling for SK2 (10 nm, red arrowheads) and mGlu_1α_ (5 nm, blue arrowheads; panels **C,D**), and double labeling for mGlu_1α_ (10 nm, red arrowheads) and Ca_V_2.1 (5 nm, blue arrowheads; panels **E,F**). In dendritic spines of PCs, SK2 immunoparticles (red arrowheads) co-clustered with those for Ca_V_2.1 and mGlu_1α_ (blue arrows). In addition, mGlu_1α_ immunoparticles (red arrowheads) co-clustered with those for Ca_V_2.1 (blue arrowheads). at, axon terminals. Scale bars: **(A–F)** 0.2 μm.

Previous electrophysiological studies showed that the activation of SK2 channels is regulated by Ca^2+^ influx through Ca_V_2.1 channels (Womack et al., 2004), which interact directly with mGlu_1_ (Kitano et al., 2003). To assess the co-existence of the three proteins in the PC surface, we performed double-labeling experiments using antibodies raised in different host species and immunoparticles of two different sizes: 5 and 10 nm (Figure 5). Similarly to SK2, immunogold particles for Ca_V_2.1 and mGlu_1α_ were found on the P-face of dendritic shafts and spines (Figure 5), consistent with the intracellular location of the epitopes recognized by the guinea pig anti-Ca_V_2.1 and rabbit anti-mGlu_1α_ antibodies (Table 1), respectively. No labeling for Ca_V_2.1 and mGlu_1α_ was observed on the E-face or on cross-fractures (Figure 5).

From the two pools of SK2 distribution, only clustered SK2 channels are preferentially associated with Ca_V_2.1 or mGlu_1α_, while isolated SK2 channels were mostly not associated with the two proteins. This colocalization of clustered SK2 channels with Ca_V_2.1 or mGlu_1α_ was observed both at the level of dendritic spines and dendritic shaft. Immunoparticles for SK2 co-clustered with those for Ca_V_2.1 (Figures 5A,B) or mGlu_1α_ (Figures 5C,D) along the extrasynaptic plasma membrane of dendritic spines, although clusters of SK2 immunoparticles were also found isolated, not associated with Ca_V_2.1 or mGlu_1α_. We quantified this ratio and found that among 60 spines analyzed, all of them immunoreacted for SK2 and Cav2.1 or SK2 and mGlu_1α_, 4 in every 10 clusters of SK2 were associated with Cav2.1 immunoparticles and 5 in every 10 clusters of SK2 were associated with mGlu1α immunoparticles. Similarly, mGlu_1α_ immunoparticles co-clustered with those for Ca_V_2.1 (Figures 5E,F), although clusters of both mGlu_1α_ and Ca_V_2.1 immunoparticles were also found isolated, not associated with each other.

**Figure 6:**
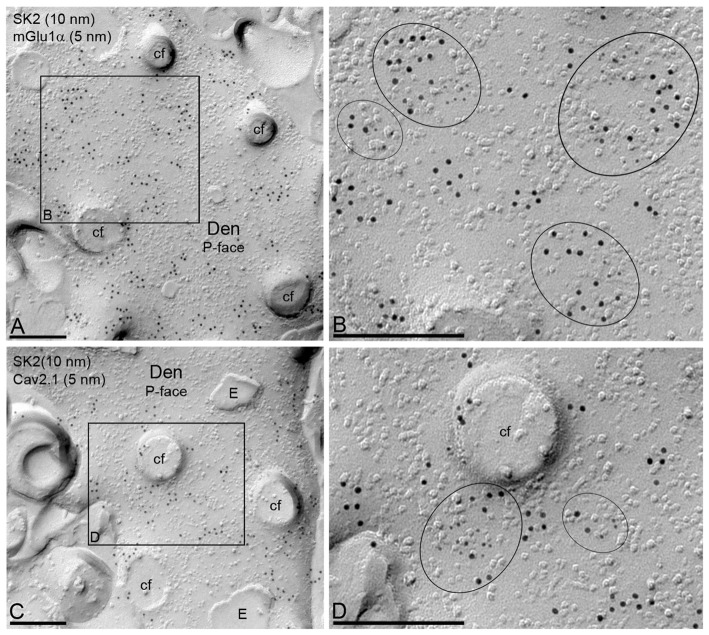
Co-localization of SK2 channels with Ca_V_2.1 channels and mGlu_1α_ receptor in PC dendritic shafts. Electron micrographs obtained in the molecular layer of the cerebellar cortex showing immunoparticles for SK2, as detected using the SDS-FRL technique. **(A,B)** Double labeling for SK2 (10 nm) and mGlu_1α_ (5 nm) showing their co-clustering in the P-face of PC dendritic shafts (Den). The black box in panel **(A)** demarcates the dendritic area shown at higher magnification in panel **(B)**. Clusters of immunoparticles for the two proteins are delineated by black ellipses. **(C,D)** Double labeling for SK2 (10 nm) and Ca_V_2.1 (5 nm) showing their co-clustering in dendritic spines of PCs. The black box in panel **(C)** demarcates the dendritic area shown at higher magnification in panel **(D)**. Clusters of immunoparticles for the two proteins are delineated by black ellipses. cf, cross-fracture of dendritic spines; E, E-face. Scale bars: **(A–D)** 0.2 μm.

In addition to dendritic spines, the co-localization between SK2, Ca_V_2.1 or mGlu_1α_ was investigated in PC dendritic shafts (Figure 6). Immunoparticles for SK2 co-clustered with those for Ca_V_2.1 or mGlu_1α_ (Figures 6A–D). However, some clusters of SK2 immunoparticles were not apparently associated with clusters of Ca_V_2.1 or mGlu_1α_ immunoparticles (Figures 6A–D). Our analysis quantifying 30 dendritic shafts, all of them double-labeled for SK2 and Cav2.1 or SK2 and mGlu_1α_, indicates that 3 in every 10 clusters of SK2 were associated with Cav2.1 immunoparticles and 4 in every 10 clusters of SK2 were associated with mGlu1α immunoparticles.

### Spatial Relationship Between SK2, Ca_V_2.1 and mGlu_1α_ in PC Spines and Dendrites

**Figure 7:**
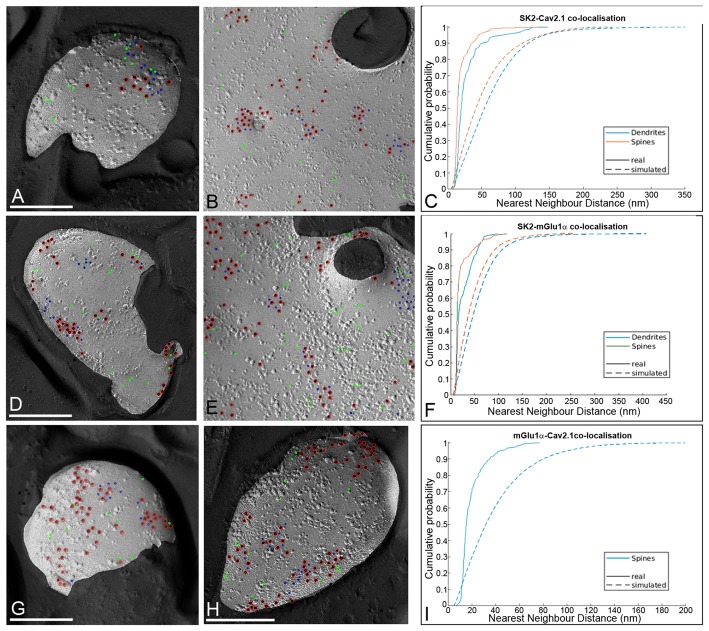
Spatial relationship between SK2, Ca_V_2.1 and mGlu_1α_ in PCs. **(A)** Example showing random simulation of immunoparticles for Ca_V_2.1 in a dendritic spine. **(B)** Example showing random simulation of immunoparticles for Ca_V_2.1 in a dendritic shaft. Red, real SK2; blue, real Cav2.1; green, simulated Cav2.1. **(C)** Cumulative probability plot of NND from SK2 to Ca_V_2.1 particles in dendrites (blue) and spines (red). Solid: real NND between SK2 and Ca_V_2.1; Dashed: simulated NND. The SK2 to Ca_V_2.1 NNDs were significantly smaller than the random NNDs in dendritic shafts and spines (*p* < 0.05, pairwise Mann–Whitney *U* test, numbers of profiles analyzed for spines and dendrites should be indicated). **(D)** Example showing random simulation of immunoparticles for mGlu_1α_ in a dendritic spine. **(E)** Example showing random simulation of immunoparticles for mGlu_1α_ in a dendritic shaft. Red, real SK2; blue, real mGlu_1α_; green, simulated mGlu_1α_. **(F)** Cumulative probability plot of NND from SK2 to mGlu_1α_ particles in dendrites (blue) and spines (red). Solid: real NND between SK2 and mGlu_1α_; Dashed NND: simulated. The SK2 to mGlu_1α_ NNDs were significantly smaller than the random NNDs in dendritic shafts and spines (*p* < 0.05, pairwise Mann–Whitney *U* test, numbers of profiles analyzed for spines and dendrites should be indicated). **(G,H)** Examples showing random simulation of immunoparticles for Ca_V_2.1 in dendritic spines. Red, real mGlu_1α_; blue, real Cav2.1; green, simulated Cav2.1. **(I)** Cumulative probability plot of NND between Ca_V_2.1 and mGlu_1α_ particles in spines (blue). Solid: real NND between mGlu_1α_ and Ca_V_2.1; Dashed: simulated. The mGlu_1α_ to Ca_V_2.1 NNDs were significantly smaller than the random NNDs in dendritic spines (*p* < 0.05, pairwise Mann–Whitney *U* test, number of profiles analyzed should be indicated).

To examine the spatial relationship of the three proteins, the NNDs between immunoparticles for SK2 (10 nm) with immunoparticles for Ca_V_2.1 or mGlu_1α_ (5 nm) were measured in our double-labeled replicas in both dendritic spines and shafts (Figures 7A–I). In dendritic spines, the mean of the NNDs between SK2 and either Ca_V_2.1 or mGlu_1α_ immunoparticles was 19.70 ± 14.80 nm (*n* = 29 images) and 21.10 ± 18.40 nm (*n* = 28 images), respectively and in dendritic shafts it was 28.14 ± 22.90 nm (*n* = 10 images) and 26.59 ± 18.20 nm (*n* = 10 images), respectively. Similarly, the mean of the NNDs between Ca_V_2.1 and mGlu_1α_ immunoparticles were 19.86 ± 11.45 nm (*n* = 27 images) in dendritic spines and 20.10 ± 11.51 nm (*n* = 26 images) in dendritic shafts. To evaluate a possible spatial association of SK2 with Ca_V_2.1 or mGlu_1α_, fitted simulations for Ca_V_2.1 and mGlu_1α_ particles were generated within the P-face areas of the spines and dendritic shafts (Figures 7A,B,D,E). The NNDs from SK2 particles to real Ca_V_2.1 or mGlu_1α_ were compared with those to the simulated Ca_V_2.1 or mGlu_1α_ immunoparticles. We found significantly smaller NNDs for the SK2 and real Ca_V_2.1 or mGlu_1α_ immunoparticles than for the randomly distributed Ca_V_2.1 or mGlu_1α_ particles in both spines and dendritic shafts (*p* < 0.05, Mann–Whitney *U* test, Figures 7C,F). In addition, to examine specific spatial association of mGlu_1α_ with Ca_V_2.1 in spines, fitted simulations for Ca_V_2.1 particles were generated within the P-face areas (Figures 7G–I). We detected significantly smaller NNDs for the mGlu_1α_ to real than simulated Ca_V_2.1 immunoparticles in spines (*n* = 45), consistent with the results described above for SK2 to Ca_V_2.1 or mGlu_1α_. Altogether, our results demonstrate that the SK2 channels, the Ca_V_2.1 channels and the mGlu_1α_ receptors show a significant association in PC spines and dendritic shafts.

### Co-localization Between SK2 and Ca_V_2.1 Channels at Presynaptic Sites

**Figure 8:**
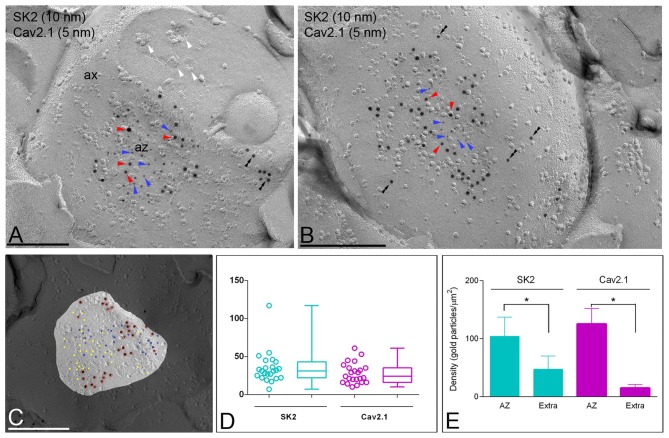
Co-localization of SK2 and Ca_V_2.1 channels in the presynaptic AZ of ATs. Electron micrographs obtained in the molecular layer of the cerebellar cortex showing the P-face and cross-fractured face of ATs (ax), which were identified by presence of synaptic vesicles (white arrowheads), as well as active zones (az) recognized by the concave shape of the P-face and the accumulation of IMPs. **(A,B)** Immunoparticles for SK2 (10 nm particles, red arrowheads) were found within the AZ co-clustering with immunoparticles for Ca_V_2.1 (5 nm particles, blue arrowheads). A few immunoparticles for SK2 were also observed along the extrasynaptic site (double arrowheads) of ATs. Scale bars: **(A,B)** 0.2 μm. **(C)** NNDs from SK2 to Ca_V_2.1 and in AZs. The NNDs from SK2 to real and simulated Ca_V_2.1 immunoparticles were generated using the same numbers of Ca_V_2.1 immunoparticles in the same compartment areas, showing no significant differences (*p* > 0.05, Kruskal-Wallis test). Insert is an example showing simulation of Cav2.1 immunoparticles in an AZ. Scale bar: 0.2 μm. **(D)** A high variability in the number of SK2 (range: 3–21) and Ca_V_2.1 (range: 4–24) immunoparticles was found at the AZs. Box chart shows 5th, 25th, 75th and 95th percentiles and median (bar). **(E)** Histogram showing the densities of SK2 and Ca_V_2.1 immunoparticles at the AZ and extrasynaptic sites of ATs. The density of both SK2 and Ca_V_2.1 immunoparticles was significantly larger at the AZs (SK2 = 104.10 ± 32.87/ μm^2^; Ca_V_2.*1* = 125.50 ± 26.61/μm^2^) than at extrasynaptic sites (Extra; SK2 = 47.24 ± 22.81/ μm^2^; Ca_V_2.*1* = 14.95 ± 5.93/μm^2^; Kruskal–Wallis test, pairwise Mann–Whitney U test and Dunns method, *p < 0.001).

Next, to determine the source of Ca^2+^ activating SK2 channels at presynaptic sites we performed double-labeling experiments for SK2 and Ca_V_2.1, known to be present in the AZ of PF terminals (Kulik et al., 2004; Indriati et al., 2013). Most SK2 immunoparticles were found in the AZs (Figures 8A,B) and less frequently outside the AZ (Figures 8A,B). The NNDs from SK2 to Ca_V_2.1 immunoparticles in the AZ was 37.5 nm and it was 83.4 nm outside the AZ. However, we found no difference (*p* = 0.84) between NNDs from SK2 to real and simulated Ca_V_2.1 immunoparticles (Figure 8C), indicating absence of significant co-clustering. Furthermore, we detected that the number of immunoparticles was highly variable, ranging from 3–21 per AZ for SK2 (Figure 8D; mean = 10.2, median = 8, interquartile range = 5.25–14.75, *n* = 40 AZ profiles from three animals) and from 4–24 per AZ for Ca_V_2.1 (Figure 8D; mean = 12.05, median = 11, interquartile range = 8.25–16.00, *n* = 40 AZ profiles from three animals). The density of SK2 and Ca_V_2.1 at the AZ was 104.10 ± 32.87 immunogold/μm^2^ and 125.50 ± 26.61 immunogold/μm^2^, respectively (Figure 8E). Low densities of SK2 (47.24 ± 22.81 immunogold/μm^2^) and Ca_V_2.1 (14.95 ± 5.93 immunogold/μm^2^) were in extrasynaptic sites of ATs (Figure 8E). Altogether, these data indicate accumulation of both SK2 and Ca_V_2.1 channels in the AZ.

## Discussion

A large body of experimental data has revealed the existence of ion channel-mediated currents in different subcellular compartments in PCs, but less information is available about the subcellular localization and density of the underlying channel subtypes/subunits. The present study provides the 2D distribution of SK2 channels along the surface of PCs and their coupling with proteins that mobilize Ca^2+^, required for SK2 activation. In the present study, we show that SK2 is restricted to somato-dendritic domains of PCs, with the highest density in PC spines. We also provide the first direct evidence that SK2 is absent from the postsynaptic density of excitatory PF-PC synapses, and always distributed in the extrasynaptic plasma membrane. Using Co-IP experiments, we show that SK2, Ca_V_2.1 and mGlu_1α_ form macromolecular complexes and using the highly sensitive SDS-FRL technique we demonstrate that the three proteins clustered together in the extrasynaptic plasma membrane of both dendritic spines and shafts in the nanodomain range (<50 nm NND). Finally, our data also show that SK2 channels can be distributed presynaptically in the AZ of ATs, presumably PF terminals, where they co-localize, although they do not co-clustered with Ca_V_2.1 channels in the nanodomain range. Our findings suggest the existence of a novel way of SK2 activation taking place outside synaptic sites that requires Ca_V_2.1 and mGlu_1α_ in dendritic domains to influence different aspects of the PC physiology.

### Distribution of SK2 Channels in PCs

The SK2 is the most abundant SK channel in the cerebellum and expressed in PCs as reported by previous *in situ* hybridization (Stocker and Pedarzani, 2000; Cingolani et al., 2002; Sailer et al., 2002; Gymnopoulos et al., 2014) and immunohistochemical studies (Sailer et al., 2004; Ballesteros-Merino et al., 2014a). At the subcellular level, using the pre-embedding immunogold technique, we previously showed a high density of SK2 in PC spines in the adult and during postnatal development (Ballesteros-Merino et al., 2014a). Here, using more sensitive approaches we have confirmed that the highest density of SK2 immunolabeling was distributed in dendritic compartments and particularly enriched in PC spines surrounding the excitatory synapses between PC spines and PF ATs in adult animals. These results match with electrophysiological data showing that a form of intrinsic plasticity mediated by the down-regulation of SK channels is related with an enhancement of Ca^2+^ transients in PC spines and that this increase lead to a lower probability for LTP induction (Belmeguenai et al., 2010; Hosy et al., 2011). Moreover, SK2 channel regulation promotes activity-dependent plasticity of dendritic intrinsic excitability, enabling PCs to adjust the properties of dendritic processing (Ohtsuki et al., 2012).

Our immunoelectron microscopic study revealed two distinct SK2 distribution patterns in the somato-dendritic compartments of PCs. Most immunoparticles for SK2 were clustered in the plasma membrane and a few of them were scattered or isolated. This characteristic distribution pattern has also been observed for SK2 in developing PCs using 3D reconstruction of PC dendrites and spines (Ballesteros-Merino et al., 2014a), as well as for SK2 and SK3 in pyramidal cells and granule cells of the hippocampus (Ballesteros-Merino et al., 2012, 2014b). Thus, the formation of clustered and isolated pools of SK channels may represent a common organizational principle in the surface of central neurons. Favoring this idea, other ion channels like BK channels, GIRK channels and Cav2.1 channels, as well as receptors like GABA_B_, have been demonstrated to follow similar distribution patterns in PCs and hippocampal pyramidal cells (Kaufmann et al., 2009, 2010; Indriati et al., 2013; Luján et al., 2018). In addition, the two distinct patterns of localization seem to be developmentally regulated in a way that isolated pools are more frequently observed at early stages while clustered pools progressively increase in number and composition of immunoparticles with postnatal age (Indriati et al., 2013; Ballesteros-Merino et al., 2014a). Given the high sensitivity of the SDS-FRL technique, which is close to unity (Tanaka et al., 2005; Indriati et al., 2013), it is plausible to think that the two pools with different content of SK2 channels are not due to technical limitations. Whether switching via lateral diffusion between the two pools of SK2 channels is possible or whether they are independent of each other is unknown. However, it has recently shown that the surface distribution of NMDA receptors, which are known to interact with SK2 channels (Lin et al., 2008), is dynamically regulated through lateral diffusion, thus providing a way to alter the receptor content between synaptic and extrasynaptic membranes (Ladépêche et al., 2014). Future studies can establish whether SK2 channels follow similar lateral diffusion in the plasma membrane.

### Non-uniform Distribution of SK2 Channels in the Surface of PCs

There is an increasing interest about revealing the differences in density of ion channels in distinct axo-somato-dendritic domains of central neurons. This is because virtually every neuron expresses several types and subunits of ion channels, whose distribution and density over the axo-somato-dendritic plasma membrane noticeably affect the function of neurons (Luján, 2010; Luján and Aguado, 2015). The density of ion channels has been studied using electrophysiological techniques (Stuart et al., 1997; Migliore and Shepherd, 2002; Johnston et al., 2003), but dendritic patch recordings are difficult to perform in small spiny branchlets and in dendritic spines. Immunoelectron microscopy offers a technical advantage to study channel distribution and density in all neuronal compartments. Thus, using the highly sensitive SDS-FRL technique we could explore how SK2 density compares between AIS, soma, main dendrites, spiny branchlet dendrites and dendritic spines of PCs. The data revealed that immunoparticles for SK2 were present along the complete axo-somato-dendritic axis of PCs, but in addition SK2 can be selectively targeted to a specific neuronal compartment at a significantly higher density. Thus, the density of SK2 immunoparticles increased significantly from the soma to dendritic spines. At approximately the same distance from the soma, significant differences were observed between the main dendrites, spiny branchlet, or dendritic spines. This non-uniform distribution pattern may help significantly to changes that determine the properties of SK2 channels and thereby affect cerebellar function (Hosy et al., 2011). Consistent with our immunoelectron microscopic data, non-uniform distribution of ion channels has been reported for members of the voltage-gated K^+^ channels (Kerti et al., 2012; Kirizs et al., 2014), G protein-gated inwardly rectifying K^+^ channels, including the GIRK1, GIRK2 and GIRK3 subunits (Fernández-Alacid et al., 2011; Kirizs et al., 2014), Ca^2+^-activated K^+^ channels, including BK, SK2 and SK3 channels (Ballesteros-Merino et al., 2012, 2014a,b), Na^+^ channels (Martínez-Hernández et al., 2013) and Ca_V_ channels (Parajuli et al., 2012; Indriati et al., 2013). However, not all ion channels follow this non-homogeneous pattern, but instead they are distributed uniformly in a cell-type dependent-manner, such as Cav3.1 in thalamic relay neurons (Parajuli et al., 2010) or SK3 in granule cells (Ballesteros-Merino et al., 2014b).

It is also important to note that we did not find differences in the SK2 density in the dendritic compartments between the inner 1/3 and outer 2/3 of the molecular layer, which represents the domains where stellate cells and basket cells, respectively, are located and establish their inhibitory contact with PCs (Altman and Bayer, 1997). This finding was not particularly surprising because SK2 channels were associated with glutamatergic synapses (Ballesteros-Merino et al., 2014a) and their establishment between PF terminals and PC spines takes place all along the whole dendritic tree (Altman and Bayer, 1997). Our immunoelectron microscopy only shows a SK2 gradient from somata to dendritic spines. The low density of SK2 in PC somata, receiving inhibitory inputs from basket cells and the high density in dendritic spines suggest that SK2 channels are distributed in a synapse-dependent manner, consistent with data described in the hippocampus (Ballesteros-Merino et al., 2012, 2014b).

### Coupling of SK2 Channels to Different Proteins Mobilizing Ca^2+^ in PCs

The coupling of SK channels to specific ion channels can be accomplished by localization of the SK channels close to the proteins that mobilize Ca^2+^ within the cellular domain in which the Ca^2+^ increases locally ion channel opening. One of the ion channels that mobilize Ca^2+^ in all brain regions is the NMDA receptor. For instance, in spines of CA1 pyramidal cells SK2 channels are activated by Ca^2+^ influx through NMDA receptors that are closely positioned in PSDs to modulate synaptic responses (Ngo-Anh et al., 2005; Lin et al., 2008). In dopaminergic neurons of the VTA inhibition of SK3 at synaptic sites enhances NMDA-mediated currents and facilitates bursting (Soden et al., 2013). In the amygdala, NMDA receptors activate SK channels and shunts excitatory postsynaptic potentials (Faber et al., 2005). However, our high-resolution immunoelectron microscopic study using two different immunogold techniques shows that SK2 channels are not distributed along the PSD in PC dendritic spines. SK2 channels were distributed either at the edge of excitatory synapses or along the extrasynaptic plasma membrane of the PC spines. Consistent with this distribution pattern, a recent study has demonstrated that an exclusively non-synaptic, SK2 channel-dependent mechanism in PCs regulated spike pauses in PCs (Grasselli et al., 2016).

Another ion channel type mobilizing Ca^2+^ that can reliably activate SK channels is the Ca_V_ channel. In dendrites of hippocampal pyramidal cells SK2 channels are activated by Ca^2+^ influx through L-type Ca_V_ channels to influence dendritic excitability (Marrion and Tavalin, 1998). In thalamic nRt neurons, dendritic SK2 channels associate with T-type Cav channels and SERCA pumps to regulate oscillatory dynamics related to sleep (Cueni et al., 2008). In the cerebellum, SK channels are activated by P/Q-type (Cav2.1) channels to control spontaneous firing in PCs (Womack et al., 2004). Consistent with this functional coupling, a proteomic approach showed that those Ca_V_ channels form macromolecular complexes with SK channels (Fakler and Adelman, 2008).

Another group of molecules controlling cytosolic Ca^2+^ transients is Group I mGlu receptors. They stimulate phospholipase C (PLC), triggering Ca^2+^ release from internal stores following activation of IP_3_ receptors (Ferraguti and Shigemoto, 2006). In the hippocampus, we have recently demonstrated that SK2 channels and mGlu_5_ receptors co-assemble and bidirectionally regulate its activity. Therefore, activation of Group I mGlu_5_ receptors mobilizes intracellular Ca^2+^, which is required to gate the SK2 channel, whereas the use of apamin to block SK2 channels limits mGlu_5_ receptor signaling (García-Negredo et al., 2014). In the cerebellum, however, PCs do not express mGlu_5_ receptors but instead they contain high levels of the mGlu_1α_ receptor, a large splice variant of mGlu_1_ subtype (Masu et al., 1991; López-Bendito et al., 2001). Interestingly, scattered large-conductance Ca^2+^-activated K ^+^ (BK) channels were also described to colocalize with mGlu_1α_ receptors in PCs (Kaufmann et al., 2009). However, clustered BK channels were elegantly reported to be associated with subsurface membrane cisterns (Kaufmann et al., 2009), which represent subcompartments of the endoplasmic reticulum enriched in IP_3_ receptors and SERCA pumps, thus mobilizing Ca^2+^ (Blaustein and Golovina, 2001). Here, we did not address the possible association of SK2 with subsurface membrane cisterns of PCs, because the SDS-FRL technique only allowed us to analyze the organization of proteins along the neuronal surface. However, at least during postnatal development, we did not observe such type of association of SK2 channels in PCs in a previous data using a pre-embedding immungold technique (Ballesteros-Merino et al., 2014a), suggesting that SK and BK channels may organize differently in PCs.

Our study using co-immunoprecipitation techniques shows that SK2, Ca_V_2.1 and mGlu_1α_ form macromolecular complexes. A previous study showed a direct interaction and functional coupling between Ca_V_2.1 and mGlu_1α_ (Kitano et al., 2003). In agreement with these biochemical data, immunoelectron microscopy showed that two types of proteins that mobilize Ca^2+^, Ca_V_2.1 and mGlu_1α_, were found to colocalize with SK2 in the nanodomain range (<50 nm NND; Neher, 1998). Our data suggest that the close association of SK2, Ca_V_2.1 and mGlu_1α_ provides the mechanism to ensure spatio-temporal regulation of intracellular Ca^2+^.

### Presynaptic Association of SK2 Channels With Ca_V_2.1 Channels

In addition to the major somato-dendritic distribution of SK2 in PCs, we also found presynaptic labeling for this ion channel in ATs establishing excitatory synapses between putative PF terminals and PC spines. We observed a high density of immunoparticles for SK2 in the AZ of ATs and low density along the extrasynaptic plasma membrane. Interestingly, immunoparticles for SK2 were closely associated with those for Ca_V_2.1 in the presynaptic AZ. Consistent with this data, a preferential localization of Ca_V_2.1 channels at the AZ of PF terminals has been recently described (Indriati et al., 2013; Luján et al., 2018). Presynaptic labeling for SK2 and SK3 channels has been shown in the hippocampus and cerebellum (Ballesteros-Merino et al., 2012, 2014a,b), as well as in cultured hippocampal neurons (Obermair et al., 2003). However, the distribution pattern we observed in the cerebellum is different to that reported previously in the hippocampus, where SK2 and SK3 channels were mainly observed extrasynaptically and only rarely at the presynaptic membrane specialization (Ballesteros-Merino et al., 2012, 2014b). The role of presynaptic SK2 channels in the cerebellum is not known, but given the preferential localization in the AZ and molecular proximity with Ca_V_2.1 channels it is plausible to think their involvement in the regulation of neurotransmitter release.

## Author Contributions

All authors had full access to all data in the study and take responsibility for the integrity of the data and the accuracy of the data analysis. RL and YF designed the project and performed SDS-FRL immunoelectron microscopy. FC and XA performed co-immunoprecipitation analysis. JM-G and LO developed in-house software and performed computational analysis. MW and RS provided reagents. JA provided knock-out tissues and feedback on the manuscript. RL, CA, AM-B and RA-R analyzed data. RL wrote the article.

## Conflict of Interest Statement

The authors declare that the research was conducted in the absence of any commercial or financial relationships that could be construed as a potential conflict of interest.
